# Nonhuman primate genetic models for the study of rare diseases

**DOI:** 10.1186/s13023-023-02619-3

**Published:** 2023-01-31

**Authors:** Eric J. Vallender, Charlotte E. Hotchkiss, Anne D. Lewis, Jeffrey Rogers, Joshua A. Stern, Samuel M. Peterson, Betsy Ferguson, Ken Sayers

**Affiliations:** 1 University of Mississippi Medical Center, Jackson, MS USA; 2 Tulane National Primate Research Center, Covington, LA USA; 3 University of Washington, Seattle, WA USA; 4 Washington National Primate Research Center, Seattle, WA USA; 5 Oregon Health and Sciences University, Beaverton, OR USA; 6 Oregon National Primate Research Center, Beaverton, OR USA; 7 Baylor College of Medicine, Houston, TX USA; 8 Wisconsin National Primate Research Center, Madison, WI USA; 9 University of California-Davis, Davis, CA USA; 10 California National Primate Research Center, Davis, CA USA; 11 Texas Biomedical Research Institute, San Antonio, TX USA; 12 Southwest National Primate Research Center, San Antonio, TX USA

## Abstract

Pre-clinical research and development relies heavily upon translationally valid models of disease. A major difficulty in understanding the biology of, and developing treatments for, rare disease is the lack of animal models. It is important that these models not only recapitulate the presentation of the disease in humans, but also that they share functionally equivalent underlying genetic causes. Nonhuman primates share physiological, anatomical, and behavioral similarities with humans resulting from close evolutionary relationships and high genetic homology. As the post-genomic era develops and next generation sequencing allows for the resequencing and screening of large populations of research animals, naturally occurring genetic variation in nonhuman primates with clinically relevant phenotypes is regularly emerging. Here we review nonhuman primate models of multiple rare genetic diseases with a focus on the similarities and differences in manifestation and etiologies across species. We discuss how these models are being developed and how they can offer new tools and opportunities for researchers interested in exploring novel therapeutics for these and other genetic diseases. Modeling human genetic diseases in translationally relevant nonhuman primates presents new prospects for development of therapeutics and a better understanding of rare diseases. The post-genomic era offers the opportunity for the discovery and further development of more models like those discussed here.

## Introduction

Understanding the causes of rare genetic disease and developing appropriate evidenced-based treatment strategies are ongoing challenges for scientists and clinicians. Rare diseases occur, by definition, infrequently, but with an estimated 10,000 unique rare diseases their overall impact is substantial [1]. The challenges and long-lasting impacts that they present for the patients, family members, and communities can be overwhelming. Uncovering the genetic basis and molecular mechanisms of these diseases are not only important for developing effective disease treatments, but also provide opportunities to better understand human biology and fundamental processes underlying human health and development.

Animal models are important tools for the study of human genetic disease for many reasons, not the least of which is the ability to study disease progression with great control in the laboratory. This is a necessary and important precondition for understanding analogous aspects of human biology and the prevention, etiologic description, or treatment of disease. The first recognition of the processes of inheritance and developmental pattern formation occurred from research with laboratory animals [2, 3]. The models are only useful and valid, however, with a comparative (across species) understanding of genotypes, phenotypes, and crucially, the relation between them. Translational studies require nonhuman models for a variety of reasons: for elucidating genetic causation or contribution to disease or normative phenotype, for understanding interactions between genetic or epigenetic factors and disease progression, for describing genotypic contributors to pharmaceutical effectiveness or the efficacy of other treatments, and for gene editing and therapy.

Among the most important considerations is the model species chosen for study. As our closest living relatives, nonhuman primates (NHPs) are especially critical research surrogates, and this review highlights new findings and directions in NHP modeling, specifically in the genetics of disease. Phylogenetic affinities, however, are only rarely the final arbiter regarding species chosen for research. Although humans are primates, there are other important, and far more widely-used, nonhuman animal models outside this order. Indeed, it has been estimated that only 0.28% of animals used in research are nonhuman primates [4]. Primates are much more expensive animal models to develop and maintain compared to other laboratory species due to their unique husbandry requirements, limited numbers, long generation time, and ethical concerns.

## Non-Primate models of disease

Although examples of animal models of disease have historically included numerous and diverse mammalian and non-mammalian taxa, the use of rodents has increased for many decades and for obvious reasons. Common laboratory rodents (mice, rats, etc.) are small, with short generation times, and are easily maintained and bred in the laboratory. Certain manipulations, including the production, maintenance, and use of specific genetic strains and the productions of particular variations, are often straightforward, allowing exquisite control of variables. This includes the development of “humanized” rodents with genes, cells, or tissue grafted from its namesake [5]. Also, their phylogenetic relationship to primates is not especially distant relative to other well-studied laboratory organisms such as zebrafish and *Drosophila* [6].

A detailed look at rodent genetic models is beyond the scope of this review. In short, much is known about rodents, and their contribution to genetically-focused biomedical advances are considerable [reviewed in 7, 8], particularly in their utility for genetic manipulation and rapid breeding. In some cases, however, the very characteristics that help make common laboratory rodents ubiquitous in experimental settings are the ones that most clearly delineate limitations as models for human beings. Their small size can present difficulties regarding the procedures that can be performed and/or translational interpretation; for example, low blood volumes and diminutive organs in rodents limit gene therapeutic and surgical approaches to pathologies such as hemophilia and retinal degeneration [9]. Small size is also coupled with a litany of differences in physiology and metabolism [10, 11]. Excepting outbred and wild strains, genetic disease investigations often require a priori hypotheses and manufactured variation, and are highly targeted, not encompassing the normal, associated variation that characterizes human population [12]. This tight control over variation offers power in experimental design, but lacks the context of inter-individual variation seen in human clinical cases.

A number of larger, non-primate mammalian models have been developed including sheep, pig, cat, and dog, often during the process of line breeding and other selective husbandry practices. While more difficult and costly to house and breed when compared to rodents, their advantages include larger organ sizes, tissues that are more easily accessible, larger blood volumes, and in some cases otherwise more humanlike anatomy or physiology. In addition, they can easily be examined as individuals with varied genotypes, phenotypes, clinical histories, prognoses, and treatment options, and examined over long durations, as are human patients [9]. In the case of domestic animals, a large number are potentially available for screening of genetic variations associated with human pathology. For example, sheep models exist for a wide range of inherited medical conditions, including visual disorders such as heritable cataracts and achromatopsia, blood and connective tissue disorders, nervous system disorders such as Batten disease, and many others [13]. The discovery and investigation of various clinical conditions in dogs is facilitated by the fact that millions of companion animals are closely monitored by their owners and regularly seen by veterinarians, providing opportunity to identify various disorders [14].

## Nonhuman primate models of disease

Non-primates are, at the end of the day, non-primates. Members of the Order Primates share a last common ancestor varyingly estimated to have lived between approximately 65 and 80 million years ago [15–20] with New World monkeys diverging 40–50 million years ago, Old World monkeys 30–35 million years ago, the ape radiation 20–25 million years ago, and the separation of the African ape/human lineage from Asian apes, 15–20 million years ago. While work has been conducted with species throughout the order, the most common NHP translational genetic model is presently the rhesus macaque (*Macaca mulatta*), a medium-sized (adults ~ 6–12 kg) Old World monkey whose genome was first reported in 2007 [21] and updated most recently in 2020 [22]. Well characterized breeding colonies of specific pathogen free rhesus macaques are maintained in the US. Work with this species is supported by a publically-available, searchable, annotated database (The Macaque Genotype and Phenotype Resource, or mGAP) on genetic variants and known disease association [23]. Additional Old World monkeys important in translational genomics research include, but are not limited to, other species of macaques, e.g. cynomolgus macaques (*M. fascicularis*) and Japanese macaques (*M. fuscata*) [24], baboons (*Papio* sp.) [25], and vervet (African green) monkeys (*Chlorocebus aethiops sabaeus*) [26]. Among New World monkeys there is growing interest in common marmosets (*Callithrix jacchus*), small simians (adults ~ 300 g) which have a published whole draft genome [27] and newer annotated reference assemblies. Marmosets are notable for litters of 2–4 offspring that are hematopoietic chimeras, as outgroups for catarrhine evolutionary studies, and for their potential in genetic engineering studies [28, 29]. Regarding prosimians an ongoing initiative is investigating the translational potential of mouse lemurs (*Microcebus* spp.), which—with their small size (30–60 g) and rapid maturation (sexually mature at 6–8 months)—present some logistical advantages similar to rodents, but with some added advantages of primate physiology [30]. Each model has its strengths and limitations, and the further development of new or underutilized model species will undoubtedly prove valuable.

While all of the primate genetic models subsequently discussed in this review occur in macaques, it is important to recognize that large-scale sequencing of vervet monkeys, baboons, and marmosets would also be expected to identify functionally significant mutations and lead to new genetic models in those species. Each primate species carries its own unique array of functionally significant mutations. The current emphasis on macaques, and in particular rhesus macaques, results largely from the significant support by the NIH for expansion of breeding programs over the past several decades, especially in response to the need for macaques for HIV/AIDS studies.

NHPs, collectively, share more genotypic and phenotypic identity with humans than any other model organisms, as primates have a number of derived features, relevant to disease modeling, that differ either qualitatively or quantitatively from other mammals. Quite striking are synapomorphies related to vision and the tactile sense [31]. Eyes are large and located anteriorly, on the front of the face, providing extensive overlap of visual fields. A bony strut (postorbital bar, rare in other mammals) lateral to the eye and, in haplorrhines, an additional bony cup behind it (postorbital plate, unique to these primates) protect these vital organs. Primate optic nerve fibers cross from one eye almost equally to both the left and right brain hemispheres for processing, in contrast to other mammals, where inputs are almost wholly to the opposing side. Collectively, these features contribute to excellent depth perception, or stereoscopic vision. Trichromatic color vision, among mammals, is likewise limited to Old World monkeys, apes, and humans with phenotypic convergence in some New World monkeys as well [32]. Platyrrhine and catarrhine primates share a primate specific retinal feature called the macula [33]. This is a region of the central retina that is especially rich in cone photoreceptors, making these species valuable as models of many human visual system disorders [34, 35]. Anthropoids are particularly important for studies of macular disorders that primarily affect cone photoreceptors, or age-related macular degeneration, where rodents, dogs and other mammals cannot provide models that mimic human macular function and dysfunction as effectively as macaques or other nonhuman primates.

Primates have excellent pedal and/or manual grasping abilities; this is exemplified by an opposable thumb in most catarrhines, flat nails rather than claws on at least some digits, and ridged tactile pads and Meissner’s corpuscles on toes and fingers, which combine for an exquisite sense of touch [31]. These adaptations speak to the importance of NHPs for investigating genetic disorders that influence vision or sensorimotor function; in an evolutionary sense, they may reflect, at least in small part, the largely arboreal, branch-grasping history of members of this order [36–38].

Primate brains, when controlled for body size, are comparatively large and differ from other mammals in some key areas of neuroanatomy. These include differences in the organization of phylogenetically ancient brain structures, unique alterations in neural connections, and novel structural units [39]. The granular prefrontal (PF) cortex, other PF areas, and certain parietal and temporal areas appear to be uniquely primate, and have been suggested to facilitate rapid goal formation, remarkable utilization of relational metrics—including quantity, duration, and distance—and flexible, sometimes immediate, solving of unique and complex problems [40–43]. Compared to other mammals, primates are strongly attuned to novelty [e.g., 44, 45] and show marked within-species, inter-individual differences in both novelty response and temperament [e.g., these exhibit heritable variation in baboons, 46] as well as between-species differences. In addition, NHPs, of all nonhuman animals, show the most extensive abilities in executive function tasks such as prospective memory, self-control, metacognition, and episodic memory [47], functions mediated by prefrontal cortical networks that are greatly expanded and differentiated in primates and have unique molecular regulation compared to rodents [48–51]. This is especially relevant in that executive function deficits are oftentimes the first to be detectable in genetic disorders involving the central nervous system, or that may presage other pathologies [52–54]. NHPs are modally very social, exhibit protracted life histories including a prolonged juvenile period, and have great ecological and behavioral plasticity [55–57]. In short, NHPs are the nonhuman animals that most closely experience the world in the manner that humans do, with myriad repercussions on the detection, description, and treatment of diseases influencing sensation, perception, behavior, and cognition.

Many other features of morphology likewise render NHPs a uniquely valuable resource for genetic modeling [58, 59]. These include numerous physiological similarities to humans that are rare or absent in other mammals. Regarding reproduction, for example, this includes humanlike estradiol-luteinizing hormone feedback loops in both males and females, similar anatomical origins for sex steroids, menstruation, and a similar pattern of reproductive senescence, in at least some species [4]. NHPs such as baboons and macaques are thus preferred models for reproductive disorders [60].

## Leveraging contemporary nonhuman primate genetics for disease model development

The similarities seen between human and NHPs reflect their close genetic affinity. Recent technical advances such as next-generation sequencing have, as noted, made feasible the construction of largely complete and well-annotated primate reference genomes, and more specifically have aided the identification of species-specific genetic variation that is directly relevant to disease risk, causation, or prognosis [61]. What follows will, we hope, give strong indication of the translational advances that have stemmed, and will stem, from further detailed primate genomics research.

Recently, the reduced cost of whole genome sequencing for research colonies of rhesus macaques has facilitated an important change in the approach to and discovery of macaque models of human inherited diseases. It is now possible to perform whole genome or whole exome sequencing on nonhuman primates from research colonies that are identified with spontaneous pathologies that may be relevant to human diseases of various kinds [12, 62]. In addition, the amount of genetic variation present in rhesus macaques and other laboratory primates creates significant opportunities for novel analyses of naturally occurring genetic variation [22, 63]. As a result, we now can more readily identify new nonhuman primate models of human genetic diseases through careful monitoring for relevant pathologies combined with genomic analysis. Two complementary approaches have proven successful: a) sequencing large numbers of research primates in order to identify potentially damaging mutations in genes known or suspected to be involved in human diseases, or b) sequencing particular animals that are identified as having pathology indicative of disease, in order to identify the causative mutation(s).

A primate model of a human genetic disease need not specifically recapitulate the mutations found in human patients. A human genetic disease caused by damaging mutations in a specific gene can often be modeled by other mutations in the same gene that disrupt protein function to an equivalent degree. The disease is the result of disrupting a genetic pathway that consists of many genes working in concert, so that a human disease can be mimicked by any mutations that produce similar disruption to that pathway. Identifying dysregulation in these gene networks beyond specific pathogenic mutations can further elucidate the larger biology of the disease and provide alternative methods to approach treatment.

In addition, it is now possible to generate novel genetic models of disease in nonhuman primates using CRISPR/Cas9 methods to make specific changes in genomic DNA sequences [64]. This approach has been used to produce macaque models related to autism [65] and Parkinsonian neurodegeneration [66]. This approach opens new opportunities to generate particular mutations in specific nonhuman primate genes, and thus can quite remarkably model human genetic disorders. However, this approach entails significant cost, requires large numbers of animals to be performed successfully and can generate off-target mutations that may in some cases compromise the translational value of the model. Moreover, it requires knowledge of the disease-causing mutation in humans a priori, precluding identification of novel disease-causing factors. Thus, while genetic manipulations of primates using CRISPR-Cas9 or analogous methods will likely play a role in the future of biomedical research, the opportunity to exploit naturally occurring variation should not be neglected.

## Specific nonhuman primate genetic models of disease

In addition to experimentally induced models of disease, nonhuman primates have long been used as genetic models of diseases having significant heritable components, including when specific genetic origins are unclear in either humans or animals. This includes complex behavioral phenotypes such as anxiety [67–70], heart disease and other cardiovascular phenotypes [71–75], obesity and type 2 diabetes [25, 76], and heritable cancers [77, 78]. For some of these diseases, nonhuman primate models have led to further elucidation of specific contributing genetic factors or targets for treatments. There is a significant historical body of literature on the genetics of alcohol use disorder, particularly the *OPRM1* gene [79–82], and a more recent breakthrough in understanding the role of *NPSR1* in endometriosis [83].

**Fig. 1:**
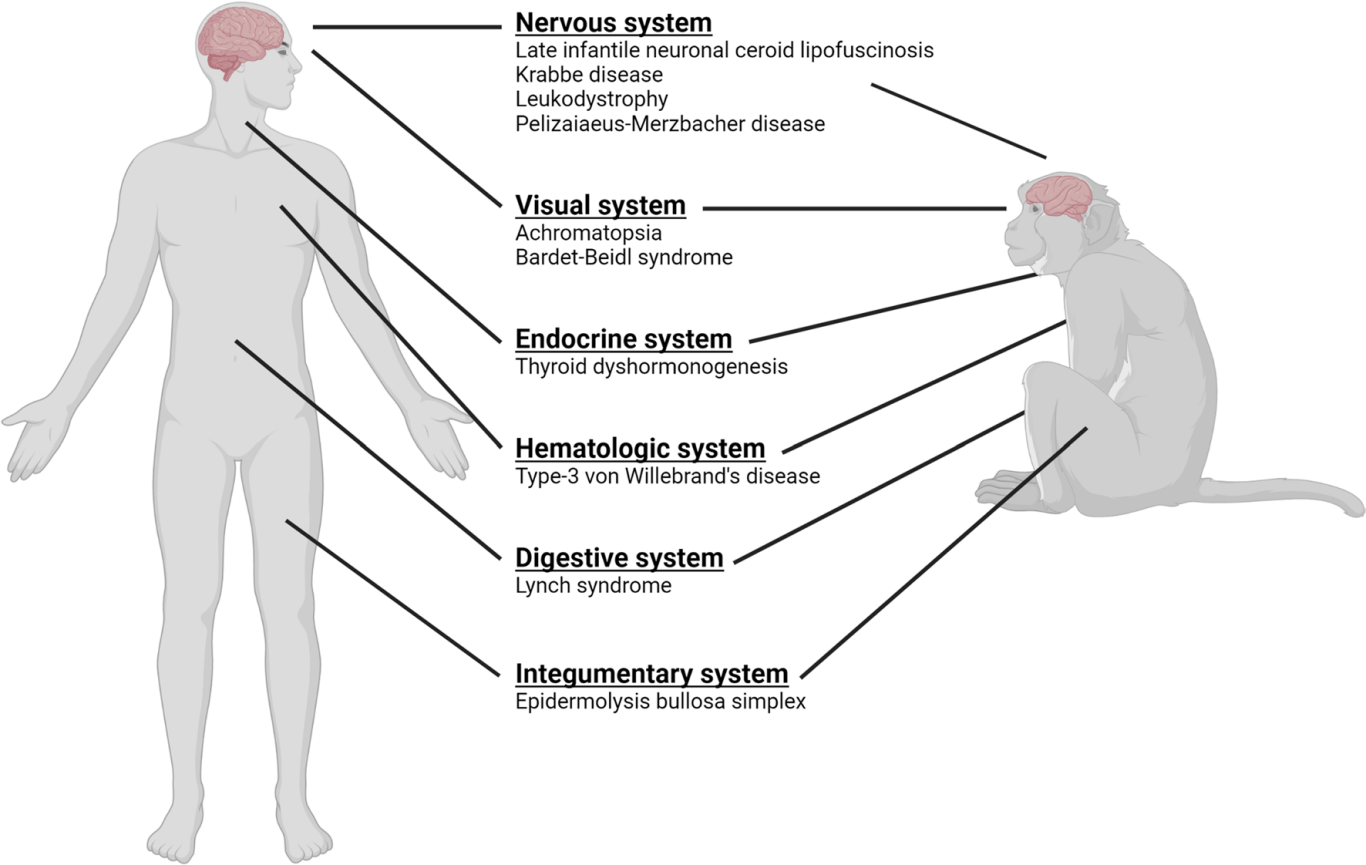
A graphical representation of select rare diseases for which genetic models in nonhuman primates have been developed. Figure created with BioRender.com

Increasingly, naturally occurring nonhuman primate models of rare human disease are being discovered and developed (Table 1). Animals born with phenotypes similar to those seen in human disease are characterized genetically and are often found to carry mutations in genes that are homologous to putative pathogenic variation in humans. The identification of disease-causing mutations present in the population can then be exploited to develop genetically-defined animal models of disease for use in preclinical studies or, alternatively, to inform breeding decisions to avoid unintentionally producing affected individuals. In the past several years, the number of NHP rare disease studies has grown significantly. The examples below illustrate the breadth and depth of models that have emerged, the pathways that have led to their development, and opportunities for future research directions. For each of these, animals or close relatives confirmed to harbor or potentially harboring disease-causing mutations are present in extant colonies. This list is not exhaustive and the details presented about disease pathologies in both humans and their NHP counterparts are brief, but it does represent a small window into the range of NHP genetic models that have been described and a guidepost for the future (Fig. 1).

**Table 1:** Select rare diseases in humans with nonhuman primate models identified

Disease	Gene	Prevalence	Inheritance	Onset
Late infantile neuronal ceroid lipofuscinosis	*CLN7*	unknown (1/200,000–1,000,000)	Autosomal recessive	Childhood
Krabbe disease	*GALC*	1–9/100,000	Autosomal recessive	Neonatal, Infancy, Childhood, Adolescent, Adult
Leukodystrophy	*CLCN2*	< 1/1,000,000	Autosomal recessive	All ages
Pelizaiaeus-Merzbacher disease	*PLP1*	1/400,000	X-linked recessive	Neonatal, Infancy
Achromatopsia	*PDE6C*	1–9/100,000	Autosomal recessive	Neonatal, Infancy
Bardet-Beidl syndrome	*BBS7*	1/100,000	Oligogenic/Autosomal recessive	Neonatal, Antenatal, Infancy, Childhood
Thyroid dyshormonogenesis	*TG*	1–9/100,000	Autosomal recessive	Neonatal, Infancy
Type-3 von Willebrand's disease	*VWF*	1/200,000–500,000	Autosomal recessive	Neonatal, Infancy
Lynch syndrome	*MLH1*	unknown (1/2,000)	Autosomal dominant	Adult
Epidermolysis bullosa simplex	*KRT5*	< 1/1,000,000	Autosomal dominant/recessive	Neonatal

## Neuronal ceroid lipofuscinosis (*CLN7*)

Neuronal ceroid lipofuscinoses (NCLs) are a collection of rare, recessive neurodegenerative diseases that typically emerge in early to middle childhood. Commonly referred to as Batten Disease, symptoms include the progressive loss of vision, speech, motor control, and cognitive skills, ultimately leading to premature death. The neuropathology of affected individuals revealed the hallmarks of lysosomal dysfunction, including the abundant accumulation of intracytoplasmic, autofluorescent lipopigment throughout the central nervous system (CNS), with associated neural inflammation and degeneration [84–87]. NCLs are autosomal recessive disorders linked to fourteen different genes *(CLN1-14)*, each encoding a lysosomal protein, endoplasmic reticulum membrane protein or a protein associated with vesicular membranes [88]. CLN7 disease specifically is associated with nonsense, missense and splice-junction mutations in the *CLN7/MFSD8* gene, which encodes a transmembrane transport protein located in the lysosomal membrane [89].

A spontaneous model of CLN7 disease was identified in a Japanese macaque *(M. fuscata)* breeding colony at the Oregon National Primate Research Center (ONPRC). Affected animals displayed progressive neurological deficits, including visual impairment, tremor, ataxia and imbalance. Imaging and functional studies revealed that CLN7 macaques have measurably reduced retinal thickness and retinal function within the first year, with profound cerebral and cerebellar atrophy progressing over five to six-years. Histological analyses detected an accumulation of highly autofluorescent storage material in cerebral, cerebellar and cardiac tissue, as well as significant degeneration of neurons. Post-mortem brain weights were 28% below average of age-matched, healthy individuals. A homozygous, single base pair deletion within *CLN7* exon 8 (c.769delA; p.Ile257LeufsTer36), was identified in five affected individuals, confirming the diagnosis [24]. To date, nine CLN7 model animals have been identified in the ~ 300 member *M. fuscata* colony.

## Krabbe disease (*GALC*)

Krabbe disease, also known as globoid cell leukodystrophy (GLD), is an autosomal recessive lysosomal storage disease associated with demyelination in the central and peripheral nervous systems. Patients with ‘classic’ or ‘infantile’ disease typically present with notable spasticity and developmental delay within the first 6 months of life, and patients typically succumb to death by 2 years of age [90]. Later onset disease, classified as ‘late infantile’ presenting at 6 months-3 years, and ‘juvenile’ presenting at 3–8 years of age, have slower disease progression and exhibit a range of symptoms that can include vision loss, cognitive decline, seizures, hypotonia, ataxia or spastic paraplegia. Disease severity is variable even within families [91]. Imaging and post-mortem studies of classic cases identify cerebral and cerebellar demyelination, as well as multinuclear (globoid) macrophages in the white matter [92]. In 1971, an association was reported between beta galactocerebrosidase deficiency and the morphologic characteristics of Krabbe disease [93]. This association was supported by the subsequent discovery of a homozygous nonsense mutation in the beta-galactocerebrosidase *(GALC)* gene in a patient with ‘classic’ Krabbe disease [94], which was followed by numerous reports of missense, insertion, and deletion variants in the *GALC* gene of Krabbe disease patients [90].

**Fig. 2:**
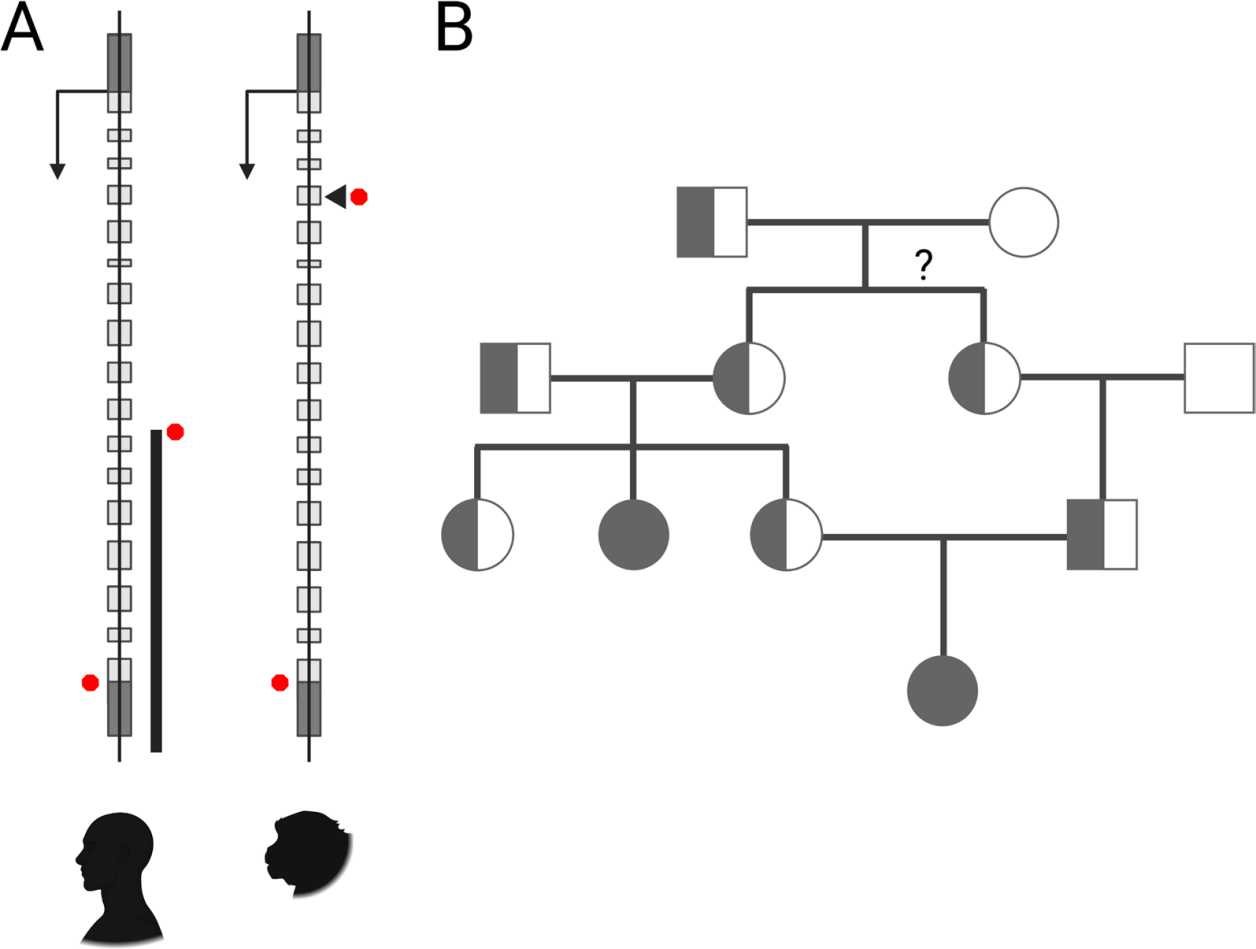
The development of a nonhuman primate model of Krabbe disease. **A** Among other pathogenic mutations, a deletion in humans eliminates the 3’ region of *GALC*, in rhesus macaques a 2 base pair deletion in exon 4 creates a frameshift and truncated protein in much the same manner. Wild-type initiation and termination positions are shown to the left of the exon model while causative mutations resulting in truncated transcripts are shown to the right. **B** A pedigree focused on the initial affected individuals (solid gray) identified carriers (half-gray) of the causative genetic mutation. Figure created with BioRender.com

A naturally occurring rhesus macaque model of Krabbe disease was discovered at the Tulane National Primate Research Center (TNPRC) more than two decades ago [95] (Fig. 2). The model was identified following the death of infant at 2 weeks of age, which had a neuropathology similar to human Krabbe disease [96]. Low GALC activity was measured in the infant’s mother, and cDNA sequencing revealed a homozygous, two base pair deletion in *GALC* exon 4 of the affected individual (c.387delAC; p.Leu130HisfsTer15) [95]. Subsequent breeding of the Krabbe disease carriers enabled additional characterization of the model. Longitudinal studies identified clinical signs of muscle tremors of head and limbs, hypertonia, progressive difficulty ambulating, ataxia, hypermetria, proprioceptive deficits, and respiratory abnormalities. At necropsy, microscopic analysis revealed a striking lack of myelin in the peripheral and central nervous system, and the cerebral, cerebellar, and spinal cord white matter was infiltrated with multinucleated globoid cells [97, 98]. The utility of this macaque model was demonstrated by a study exploring the therapeutic potential of intracranially administered mesenchymal stem cells (MSCs) to treat early onset Krabbe disease. The treatment elicited transient improvements in coordination, ambulation, cognition, and large motor skills, providing preliminary support for further study of MSCs for the treatment of lysosomal storage diseases [99].

## CLCN2-related leukodystrophy (*CLCN2*)

CLCN2-related leukoencephalopathy (CC2L) is a rare autosomal recessive, neurological disease, previously called Leukoencephalopathy with Ataxia (LKPAT). The discovery of loss of function mutations in the chloride-gated voltage channel 2 *(CLCN2)* gene among six unrelated patients clarified the molecular basis of the neurological features [100]. The spectrum of symptoms range from childhood onset with mild ataxia, learning disabilities, and headaches to adult onset with mild ataxia and decreased vision. Infertility has been reported in two adult males [100, 101]. MRI analysis identifies white matter abnormalities in CC2L cases. Affected individuals have decreased apparent diffusion coefficient values in the posterior limbs of the internal capsules, middle cerebral peduncles, pyramidal tracts in the pons, and middle cerebellar peduncles. The findings indicate myelin microvacuolation within certain brain regions [100].

A novel leukodystrophy case was identified in a juvenile rhesus macaque born at the ONPRC. Close observation identified three short seizure type periods and repeated episodes of hand and arm tremors. A “star gazing” behavior, typical of macaques with impaired vision, was also noted. At necropsy, primary findings included laminar cerebral neuronal necrosis, and diffuse marked vacuolation of the central nervous system white matter. Pedigree analysis identified the case to be the product of a consanguineous mating, suggesting a possible genetic link to the neurological symptoms. Subsequent genomic sequencing of the proband revealed a homozygous *CLCN2* missense mutation (c.1412G > A; p.Arg471His) that is identical to human ClinVar allele 214,433 (p.Arg471His) and associated with CC2L [102]. The same macaque allele has been detected in both the Wisconsin National Primate Research Center (WNPRC) and ONPRC breeding colonies, and has an overall a minor allele frequency of 0.0098 in the mGAP database. Taken together, the clinical, histological and genetic findings suggest this to be the first identified case of nonhuman primate CC2L.

## Pelizaeus–Merzbacher disease (*PLP1*)

Pelizaeus–Merzbacher disease (PMD) is the most common leukodystrophy associated with hypomyelination, affecting approximately 1 in 400,000 individuals [103]. PMD symptoms range in severity, with mild cases developing spastic paraplegia over time, and severe cases presenting with hypotonia, nystagmus, respiratory distress, and stridor shortly after birth [104]. PMD is caused by mutations in the proteolipid apoprotein *(PLP1)* gene, an X-linked gene that encodes a lipid binding protein that is reported to stabilize myelin [105, 106]. While *PLP1* gene duplications, deletions and point mutations have been linked to PMD, the most severe cases have frequently been associated with missense mutations in exons 2 and 4 [107].

A rhesus macaque model of PMD was recently identified at the ONPRC, following the clinical and genetic analysis of three male neonates exhibiting neurological deficits [108]. The infants displayed profound intention tremors, head bobbing, nystagmus and reduced respiratory capacity. Histopathological analyses of brain tissue obtained following necropsy revealed a CNS dysmyelinating disorder, as initially assessed by Luxol fast blue staining. Immunohistochemical analysis identified, a complete lack of the myelin protein PLP throughout the corpus callosum, a marked reduction in the expression of myelin binding protein (MBP), and a lack of clear myelinated structures. Pedigree analysis identified all three males to be related, and genomic sequencing identified a missense mutation within the *PLP1* gene (*PLP1*: n.682 T > C; p.Cys228Arg) [108]. To date, the *PLP1* missense mutation has only been detected at the ONPRC and has a minor allele frequency of 0.0004 among the 2,054 rhesus macaques reported in the macaque Gentoype and Phenotype database (mGAP) [23].

## Achromatopsia (*PDE6C*)

Achromatopsia, or rod monochromacy, is characterized by the loss of macular function critical for high acuity vision, as well as color perception. A progressive disorder, initial vision problems manifest in childhood with a gradual decline until adulthood by which time cone photoreceptors are lost completely. People with achromatopsia are also photophobic due to their dependence on rod photoreceptors. This disease is genetically heterogenous in humans with mutations inherited in an autosomal recessive manner.

Colony staff at California National Primate Research Center (CNPRC) identified two juvenile rhesus macaques that displayed evidence of visual impairment. Whole genome sequencing of these animals revealed that both are homozygous for a missense mutation in the gene *PDE6C* that inactivates the catalytic domain of this enzyme [35]. Ophthalmic examinations showed that the affected macaques have essentially normal function of their rod photoreceptors, but little or no cone photoreceptor function. This is logical because *PDE6C* is expressed in cone but not rod photoreceptors; loss of cone function will impair high acuity vision and color perception but preserves peripheral vision. *PDE6C* is one of six genes associated with achromatopsia in humans [109]. Macaques with *PDE6C* mutations also show evidence of difficulty in bright light. Subsequent genotyping of additional CNPRC rhesus macaques has identified a number of heterozygous carriers, and a breeding program has been established to produce additional homozygotes.

## Bardet-Beidl syndrome (*BBS7*)

Bardet-Biedl Syndrome (BBS) is a complex disorder exhibiting variable phenotypic expression, but generally including retinal degeneration, obesity, and kidney dysfunction along with various other symptoms. Although syndromic with broad systemic effects, the most common indication of the disease is severe vision loss during childhood. BBS is a ciliopathy affecting cell structures involved in cell–cell communication and development. Approximately 1 in 250,000 individuals have BBS and there is currently no cure for the disease. More than 20 different genes have been implicated in BBS and mutations are commonly recessive and often oligogenic [110].

Several rhesus macaques from the ONPRC were recognized as having both spontaneous retinal degeneration and kidney disease [111]. Initially, a female rhesus macaque presented with significant visual impairment and multiple structural and histological problems affecting the kidneys. Genetic analyses found that this animal was homozygous for a single base deletion in exon 3 of the gene *BBS7*. Analyses of other rhesus macaques heterozygous and homozygous for the exon 3 deletion confirmed the genetic association and diagnosis as Bardet-Biedl Syndrome. The additional genetically validated and affected animals similarly displayed the range of deficits described in human BBS7 disease. Moreover, the retinal pathology involved loss of function of photoreceptors and closely parallels the disease progression described in human Bardet-Biedl Syndrome [111, 112].

## Thyroid dyshormonogenesis (*TG*)

Thyroid dyshormogenesis occurs when the pathway leading to the synthesis of the hormonally active iodothyronines, T4 and T3, is defective. This leads to hypothyroidism and goiter. The downstream effects of congenital hypothyroidism are numerous including poor growth and delayed development particularly when untreated.

Genetic mutations leading to defects in thyroid hormone synthesis account for 15–20% of all cases of congenital hypothyroidism in humans, representing approximately 1:25,000 neonates [113]. The genes most commonly affected are *DUOX2*, *SLC5A5*, *TG*, and *TPO*. Mutations, almost always inherited in a recessive manner, result in abnormally low thyroid hormone levels [114].

Dyshormonogenetic goiter due to a defect in *TG* has been recognized in rhesus macaques at ONPRC. The initially identified animal was euthanized due to musculoskeletal abnormalities. The thyroid gland was markedly enlarged, pink, and fleshy. Microscopically, the follicular epithelium was hypertrophic and hyperplastic with diffuse colloid atrophy. The long bones exhibited epiphyseal immaturity and dysplasia, typical of congenital hypothyroidism. This animal was homozygous for a frame-shift deletion in the *TG* gene (c.5513_5514delAA, p.Lys1838fs). Two additional stillborn animals were identified with similar gross and microscopic appearance of the thyroid gland; one was confirmed as homozygote and the other was the offspring of a heterozygote dam. Of note, phenotypically similar cases of hypothyroidism associated with congenital goiters were previously described in this colony [115]. The three affected animals were offspring of the same parents. Two of the affected animals lived to adulthood; the other was stillborn following prolonged gestation. The adult animals exhibited abnormal facial features suggestive of delayed bone growth. The underlying genetic defect was not identified.

## Type 3 von Willebrand’s disease (*VWF*)

Von Willebrand’s disease (VWD) represents the most commonly inherited disorder of human coagulation and afflicts up to 1% of the population [116]. It occurs in multiple primary types, with type 1 representing reduced quantity of Von Willebrand’s factor (VWF), type 2 representing reduced function of VWF, and type 3 representing an absence of VWF [116]. Each of these types can lead to bleeding due to VWF’s role in platelet adhesion and aggregation. There are over 1000 described mutations that result in VWD and those are most commonly associated with type 2 or 3 disease. Type 3 VWD is inherited in either a recessive or co-dominant fashion with absence of VWF owing to mutations in the VWF gene that render the individual to have no circulating VWF [116].

Type 3 VWD has been described in a family of rhesus macaques where one young monkey had no measurable VWF activity [117]. This disease was identified at the New England Primate Research Center in an individual that was found to be persistently bleeding despite only minor injuries at a young age [117]. The disease was found to be familial on screening of nuclear family members where reduced VWF levels were identified in other adults macaques [117]. These findings are typical in type 3 VWD in humans where heterozygous individuals may have reduced VWF but do not spontaneously bleed [116, 117]. This rhesus model of type 3 VWD was proposed to be autosomal recessive in its pattern of inheritance, however no genetic study of mutations underlying this disorder in this pedigree was performed [117].

## Lynch syndrome (*MLH1*)

Lynch Syndrome is the most common form of hereditary colorectal cancer. The disease is caused by inheritance of damaging mutations in one of four DNA mismatch repair genes that code for proteins essential to the DNA repair mechanisms in human cells. People who inherit a dysfunctional copy of one of these four genes (*MLH1*, *MSH2*, *MSH6* or *PMS2*) are at dramatically increased risk for colorectal cancer as well as tumors of the ovary, endometrium, stomach and other organs. More than one million people in the United States carry a Lynch Syndrome mutation that raises their cancer risk [118] with mean age of onset in their 40’s. In addition to mutations leading to tumorigenesis, Lynch Syndrome patients also experience insertion/deletion mutations in microsatellite repeats within protein coding genes. These indel mutations generate novel peptide sequences (neoantigens) that can be used to stimulate the native immune system and thus to attack tumor cells.

More than 60 cases of spontaneous colorectal cancer have been identified in rhesus macaques at the Keeling Center for Comparative Medicine and Research, part of the University of Texas MD Anderson Cancer Center [78]. The tumors observed in these animals are generally located in the ileocecal junction, proximal colon or cecum, highly reminiscent of human Lynch Syndrome tumors. In addition, significant histological similarities were identified, and immunohistochemistry showed that the tumors generally lacked expression of MLH1 and PMS2 proteins [78]. Subsequent DNA sequencing showed that macaques suffering these tumors had significantly elevated frequencies of a stop codon in *MLH1* (c.1029C < G, p.Tyr343Ter). Furthermore, carriers of this mutation showed substantial instability in microsatellite sequences across the macaque genome, a widely used indicator of Lynch Syndrome pathology in humans. Following these initial genomic analyses, gene expression analyses have shown that the macaque tumors exhibit transcription profiles very similar to those in human Lynch Syndrome tumors [119].

## Epidermolysis Bullosa simplex (*KRT5*)

Epidermolysis bullosa simplex (EBS) is one of the most common genetic bullous skin diseases. It is characterized by the separation of the skin at the basal keratinocytes region after trauma and blister formation. The prevalence is estimated to be 1 per 25,000–50,000 births, and depending on the mutation, can follow either recessive or dominant inheritance [120]. EBS is a heterogenous group of diseases, which range from localized to severe effects. Mutations in keratin 5 (*KRT5*) and keratin 14 (*KRT14*) genes account for the majority of EBS cases [121]. Phenotype–genotype analysis of patients has shown that mutations that disrupt the central alpha-helical rod of the keratin protein are associated with a more severe disease phenotype [122].

A natural rhesus macaque model of EBS was recently reported at the ONPRC. Two stillborn macaques were initially recognized as being likely EBS cases based on the appearance of widespread sloughing of skin at delivery. Microscopic findings in both animals included multifocal to coalescing, variably sized intraepidermal clefts, with prominent basal cell vacuolation and fragmentation. Retrospective DNA sequence analysis determined that the two cases were both homozygous for a 34 bp insertion within *KRT5* exon 5 (p.Lys363fs), predicted to disrupt the 4th coil of the alpha-helical rod. At the time of discovery, the mGAP database included 278 genomes and the *KRT5* mutation occurred at 0.0054 frequency, with no other homozygotes detected. Six asymptomatic individuals with heterozygous KRT5 insertion mutations were subsequently identified, based on pedigree relationship to the focal cases [123].

## Implications for future work

Genetic diseases in humans are also present in nonhuman primates. While this simple fact has been long recognized, it has been difficult to take advantage for developing research models. The spontaneous and irregular emergence of animals with disease phenotypes often could not be duplicated, particularly for diseases that caused premature death or diminished reproductive capacity. Advances in our fundamental understanding of primate genomes, the reduced cost of whole genome sequencing, and the subsequent large-scale identification of nonhuman primate genetic variation have not only allowed for a better understanding of the molecular underpinnings of disease in nonhuman primates, but they also allow for greater control over the production of these models.

The identification of disease-causing mutations in nonhuman primates is an important advance for biomedical research; it allows for the purposeful breeding of animal models to study human disease. These models often faithfully and accurately recapitulate human disease in both presentation and etiology, allowing for the discovery of relevant biomarkers and the pre-symptomatic or longitudinal study of disease pathology. The full range of state-of-the-art biomedical techniques, including advanced imaging, electrophysiology, auditory, optical, and cognitive measures can be monitored. Post-mortem gross and microscopy histology can be used to study disease-associated molecular and cellular changes with minimal confounding factors. Importantly, the relevance of the findings are directly translational to the human condition.

One of the most exciting aspects of the genetically parallel, NHP disease models are the associated opportunities to develop and test promising approaches to the treatment or cure of these human diseases. Development of treatments in utero or otherwise prior to symptom emergence is now feasible in ways that were previously inaccessible. Genomic medicine approaches, including protein replacement, gene silencing or editing, and stem cell therapies can be evaluated in these translational models to insure efficient delivery, distribution, and longevity. Pre-clinical testing in an anatomically and physiologically relevant, large animal model will also be key for optimizing the efficacy, safety, and specificity of such treatments. The recent advances in NHP rare disease model development are both timely and critical as these emerging genomic-based approaches are likely to be the best, if not only, way to effectively treat many of these debilitating or fatal rare diseases.

## Conclusions

Nonhuman primates represent an important model organism for biomedical research. Importantly, they can also serve a valuable role as genetic models of disease, including rare disease. Taking advantage of the genetic similarities between humans and NHPs and the recent advances in next generation sequencing technology that have allowed for increasingly comprehensive catalogs of NHP variation, new models of human rare disease are emerging. These offer the possibility for new therapeutic development and understandings of disease that have been previously elusive.

## Data Availability

Genomic data is available at http://mgap.ohsu.edu or from the corresponding author on reasonable request.
